# Anabolic Androgenic Steroids in Orthopaedic Surgery: Current Concepts and Clinical Applications

**DOI:** 10.5435/JAAOSGlobal-D-21-00156

**Published:** 2022-01-04

**Authors:** Alexander E. Weber, Matthew C. Gallo, Ioanna K. Bolia, Emmett J. Cleary, Todd E. Schroeder, George F. Rick Hatch

**Affiliations:** From the USC Epstein Family Center for Sports Medicine at Keck Medicine of USC, Los Angeles, CA.

## Abstract

Despite the well-documented effects of testosterone and its synthetic derivatives—collectively termed anabolic androgenic steroids (AASs)—on the musculoskeletal system, the therapeutic use of these agents has received limited investigation within the field of orthopaedic surgery. In the last 2 decades, preclinical and clinical research has started to identify promising applications of the short-term use of AASs in the perioperative period. There is evidence to suggest that AASs may improve postoperative recovery after anterior cruciate ligament reconstruction and total joint arthroplasty. In addition, AASs may augment the biological healing environment in specific clinical scenarios including muscle injury, fracture repair, and rotator cuff repair. Current literature fails to present strong evidence for or against the use of AASs in orthopaedics, but there is continuous research on this topic. The purpose of this study was to provide a comprehensive overview of the current status of AAS applications in orthopaedic surgery, with an emphasis on preclinical data, clinical studies, and future directions.

Anabolic androgenic steroids (AASs) are synthetic testosterone derivatives designed to maximize anabolic activity and minimize androgenic effects.^1^ AASs have gained considerable notoriety in the last half century, which is attributable to their illegal use in athletic competition.^2,3^ Despite their reputation, AASs have a number of therapeutic applications. The anabolic effects of AASs may play a significant role in the treatment of muscle wasting associated with severe burns and a wide spectrum of chronic diseases such as human immunodeficiency virus, cancer, renal failure, hepatic cirrhosis, pulmonary disease, and muscular dystrophy.^4,5,6,7,8,9,10^ AASs have also been studied in the context of prolonged immobilization after spinal cord injury, which is characterized by volumetric bone and muscle loss; preclinical studies have shown beneficial effects, and a clinical trial is underway to validate these promising findings.^11-13^

The use of AASs to counteract muscle and bone loss is supported by a growing by body of evidence showing a positive effect of AASs on muscle mass, strength, and bone metabolism.^14,15,16,17^ The applications of AASs related to the field of orthopaedic surgery have been historically limited. A notable exception is the use of AASs in the treatment of osteoporosis, which has been studied more extensively.^18,19,20,21,22^ Although testosterone may be indicated for men with osteoporosis, other AASs have been studied as a possible alternative, particularly in women, due to the lower risk of virilizing side effects.^23,24^ The clinical results with the use of AAS as an adjunct therapy in osteoporosis remains a subject of inquiry in both the male and female population.^24,25,26,27^ AASs have mostly been evaluated at a relatively low dose and with long-term administration in studies on osteoporosis.^4,24,25,26,27,28^ Only in the last two decades has short-term administration of AAS received investigation in orthopaedics.

Orthopaedic injury and accompanying surgery are almost always associated with the development of disuse muscle atrophy, which lengthens recovery and delays return to full strength.^29^ Disuse atrophy has been linked to negative outcomes in common orthopaedic procedures including but not limited to anterior cruciate ligament (ACL) reconstruction, total knee arthroplasty (TKA), and lower extremity fracture repair.^30,31,32,33,34^ Recent evidence suggests that the anabolic effects of AASs in muscle may be an effective method to improve postoperative recovery after such procedures.^35-37^ For example, the anabolic environment induced by AAS may also improve biological healing in specific clinical scenarios including muscle injury, fracture repair, and rotator cuff repair.^21,38-40^ Although the degree of direct muscle and bone healing from AAS administration remains inconclusive, a recent series of preclinical experiments demonstrated that AASs may halt fatty infiltration and improve healing of a repaired rotator cuff.^39-41^ This application highlights the potential of AASs to address the underlying pathophysiology in one of the most common causes of musculoskeletal pain and disability.^42,43^

To our knowledge, the last review to focus on the therapeutic applications of AAS in orthopaedic surgery was published in 2004, which was before many recent studies on the short-term use of AAS.^28^ The purpose of this present review is to provide a summary of the current status of AAS applications in orthopaedic surgery, with an emphasis on preclinical data, clinical studies, and future directions.

## Mechanism of Action and Effects of Anabolic Androgenic Steroids on Musculoskeletal Tissues

The physiologic effects of testosterone and AASs in the musculoskeletal system have been reviewed elsewhere and will not be discussed in detail.^16,44-46^ Briefly, AASs are synthetic testosterone derivates that were developed with the goals of increased androgenic potency, prolonged biologic activity, and, in some cases, oral bioavailability.^28^ Compared with testosterone, which has an anabolic: androgenic ratio of 1:1, oxandrolone, for example, has a ratio of 13:1.^46^ Although developed to maximize anabolic activity, all AASs have some androgenic effects and may result in virilizing side effects depending on the dose and duration of use.^16^

AASs are thought to exert their actions through several pathways.^46^ First, AASs bind cellular androgen receptors (ARs), which promotes gene transcription that increases protein synthesis and reduces catabolism.^47^ Second, AASs interfere with glucocorticoid receptor expression, which results in an anticatabolic effect.^48^ Third, AASs exert effects on the central nervous system—though not fully elucidated—that are reflected in the behavioral changes seen with AAS use.^46^ Finally, AASs may be converted into dihydrotestosterone by the enzyme 5‐α‐reductase; dihydrotestosterone binds with high affinity to ARs and plays a larger role in the unwanted androgenic effects of AASs.^45,49^

AASs have an important role in musculoskeletal tissue homeostasis and have been reported to influence the biology of muscle, bone, tendons, and ligaments.^50,51,52,53^ A concise summary is presented below.

## Effect of Anabolic Androgenic Steroids on Skeletal Muscles

Testosterone induces anabolic activity directly in muscle through ARs present in myocytes.^54,55^ Indirectly, testosterone mediates muscle growth via increases in insulin-like growth factor 1 and growth hormone levels.^44,55^ It has also been shown to regulate the activity of immune, fibroblast, and myogenic precursor cells, which are all involved in muscle regeneration after injury.^44,55^ Preclinical studies in rodents with AR deletion, AR overexpression, and in castrated male rats have provided valuable insights on the importance of testosterone in the development and maintenance of muscle mass.^56-58^ These findings have been also been validated clinically: testosterone replacement has been shown to increase lean body mass and muscle mass in young men and in old men with androgen deficiency.^15,17,59^ Testosterone may also increase strength in a dose-dependent manner.^15,60^

### Effect of Anabolic Androgenic Steroids on Bone

Testosterone is a key regulator of bone mass in males.^44,61^ Trabecular bone mass is mediated by the direct effects of testosterone on AR on osteoblasts, which in turn control osteoclastic resorption.^62,63^ In contrast, AR deletion studies in rodents have found that testosterone acts through an indirect mechanism to regulate cortical bone.^64^ In clinical studies, lower testosterone in older men is associated with lower bone mineral density (BMD), whereas testosterone supplementation in older or hypogonadal men can increase BMD.^65,66^ In women, estrogen is the primary regulator of bone metabolism.^44,61^ However, a study found that postmenopausal women with higher circulating testosterone levels had higher BMD, suggesting a secondary role for testosterone in regulating female bone mass that may increase in importance with aging.^67^

### Effect of Anabolic Androgenic Steroids on Tendons

A widely reported side effect of AAS use is an increased risk of tendon rupture.^68,69^ Two hypotheses are often described in the literature: (1) AASs have no direct effect on tendons but can induce muscular hypertrophy and increased muscle strength, without associated tendon strengthening, resulting in an increased risk for rupture, or (2) that AASs, in combination with physical exertion, have a deleterious effect on tendon structure and healing.^28,69^ Preclinical studies in rodents have suggested that AAS use may negatively affect collagen synthesis and matrix metalloproteinase activity, which are both involved in tendon repair and homeostasis.^70,71^ However, as a recent review noted, investigations to date have produced inconsistent results, and it is still unclear how AASs influence tendons.^69^

### Effect of Anabolic Androgenic Steroids on Ligaments

The effect of testosterone on ligament homeostasis is poorly understood. Surgical ACL specimens from young men and women have revealed that the ACL expresses ARs in both sexes, which suggests that it is a testosterone-responsive tissue.^53,72^ Furthermore, small preclinical and clinical studies have found that testosterone levels correlate with ACL stiffness, signifying a possible role in remodeling and tensile strength.^72-74^ As other sex steroids, namely estrogen and progesterone, have been shown in vitro to influence proliferation of fibroblasts and collagen synthesis in the ACL, testosterone may function similarly, but this has not been demonstrated.^75,76^ Although more work is needed to characterize the pathways through which testosterone acts on ligaments, the influence of sex steroid hormones has been proposed as one possible explanation for the sex disparity in ACL injury risk.^77^

### Side Effects of Anabolic Androgenic Steroids

The effects of AASs in other tissues contribute to their known side-effect profile, which has largely been established through observational studies and case reports of nonmedical AAS users.^28,68,78,79^ The side effects of AAS use are generally benign and reversible (eg, acne, gynecomastia, and testicular atrophy), but long-term high-dose use is associated with severe adverse effects including irreversible cardiovascular disease and hepatic dysfunction.^80,81^ AASs may also cause dose‐dependent behavioral and psychiatric effects including euphoria and aggression; long-term administration may alter dopamine, serotonin, and opioid neurotransmitter levels.^46,82^ Side effects specific to women include hirsutism, voice deepening, male-pattern baldness, and menstrual abnormalities, some of which persist even after AAS discontinuation.^83^

In prospective clinical studies, AASs have demonstrated an acceptable safety profile. Short-term use of physiologic or supraphysiologic doses of AASs in men has not been associated with serious adverse effects on lipid levels, hepatic function, hemoglobin levels, or behavior/mood.^15,84,85,86,87,88^

## Orthopaedic Applications of Anabolic Androgenic Steroids

### A. Augmented Biological Healing Environment

#### A1. Muscle Regeneration After Injury

Muscle contusion and strain injuries comprise more than 90% of all sports-related injuries.^89^ Although skeletal muscle has a robust capacity for self-repair, the severity of injury may result in delayed healing or incomplete healing that is complicated by fibrosis.^90,91^ Injury severity may also positively correlated with the duration of functional disability.^92^ Preclinical studies have shown an association between testosterone and processes implicated in muscle regeneration.^44^ However, few studies have directly evaluated the effect of AASs on muscle regeneration, and the available evidence is conflicting.^93^ Ferry et al.^94^ examined the effects of nandrolone decanoate (ND) on the effects of the soleus and extensor digitorum longus muscles after myotoxic injection. The authors found that ND increased the mass of the soleus but had no effect on the extensor digitorum longus relative to controls.^94^ In a follow-up study, the same group found that ND did not improve isometric contractile strength in either muscle at 21 days postinjury.^95^

In a rodent model of muscle contusion, Beiner et al.^96^ found that ND administration did not increase the force-producing capacity of the gastrocnemius at 7 or 14 days postinjury. Conversely, two other preclinical studies found that ND administration was able to increase muscle regeneration, which was evaluated based on the number and morphology of myofibers present.^97,98^

Differences in preclinical findings may be explained by a varying regenerative response to different modes of muscle injury (eg, toxin induced versus contusion), dose and duration of androgen supplementation, and the outcome variables. To date, no human studies have evaluated the role of androgens in promoting muscle regeneration. Additional preclinical and clinical studies will be needed to better define how testosterone and AASs modulate muscle regeneration and the dose and duration for which they should be given for potential clinical benefit.

#### A2. Fracture Repair

Failure of fracture healing occurs in up to 10% of all patients and often leads to significant patient morbidity and substantial socioeconomic costs.^99^ Despite the known effects of testosterone in skeletal homeostasis, the direct effect of testosterone and AAS on fracture healing has not been well defined.^44^ Tarsoly et al.^38^ were the first to study this in 1979, showing that hypophysectomized (ie, lacking pituitary) rats had impaired callus formation but that this effect could be attenuated with testosterone supplementation. In another rodent study, Frankle et al. compared callus formation in rats with humeral osteotomies that were treated with weekly testosterone or methenolone enanthate, which has a high anabolic: androgenic ratio.^100^ The authors found that by 4 weeks, the calcium composition of the callus was similar between rats treated with testosterone or methenolone enanthate, with both groups having significantly more calcium than controls.^100^ Somewhat surprisingly, biomechanical testing and histology did not reveal a significant difference between the three groups at any time point (up to 6 weeks postinjury).^100^

It should be noted that the two studies referenced above involve systemic administration of testosterone or AASs.^38,100^ More recent animal studies have also shown that local administration of testosterone—in the form of a testosterone loaded scaffold material—can heal critical-sized long bone defects that would otherwise not heal.^101-103^ Although these studies suggest that testosterone is effective in fracture repair, only one study to date has evaluated its efficacy.^104^ Cheng et al.^104^ compared locally delivered testosterone and recombinant human bone morphogenetic protein 2 (rhBMP-2) for healing critical-sized femoral defects in mice. Micro-CT analysis of callus formation and bone regeneration as well as histological examination of trabecular and cortical bone found that found that testosterone was as effective as rhBMP-2 in fracture healing.^104^ RhBMP-2 is FDA approved for use in the treatment of open tibial fracture and anterior spinal fusion but is expensive and is associated with a number of adverse effects.^105^ Therefore, the authors concluded that testosterone may represent an effective, cost-effective osteoinductive stimulus for fracture healing.^104^ Future studies should seek to validate these preliminary findings in larger animal models of clinically relevant scenarios of bone loss such as fracture nonunion, revision total joint arthroplasty, and pseudarthrosis of the spine.

#### A3. Rotator Cuff Repair

Rotator cuff tears are one of the most common causes of musculoskeletal pain and disability and impose significant personal and societal costs.^42,43^ Despite advances in surgical techniques, incomplete healing and retears are common postoperative complications, with retear rats of up to 40% for small and medium tears and more than 90% in large or chronic tears.^106,107^ Incomplete healing and retears impose additional burden on patients and contributes to inferior outcomes.^108^ Therefore, there is an urgent need to improve rotator cuff healing rates. The biological hallmarks of a torn rotator cuff are muscle atrophy, fatty infiltration, and intercellular fibrosis of the muscle-tendon unit and are important prognostic factors for treatment.^109,110,111,112^ Recent studies with AASs have tried to address this underlying pathophysiology.

Preliminary studies evaluating the effects of AASs on isolated rotator cuff tendon produced contradictory findings on the protective or detrimental role of AASs.^70,113,114,115,116,117^ The results of the study by Triantafillopoulos et al.,^113^ in particular, should be interpreted with caution as their group used a bioartificial tendon that produced results inconsistent with much of the available literature. However, a series of experiments involving the rotator cuff musculotendinous unit—not just the tendon itself—have provided valuable new insights on the potential role of AASs to improve rotator cuff healing.^39,41,109^

In the first experiment, Gerber et al.^109^ released the infraspinatus tendon in six sheep and monitored muscular changes for 40 weeks with CT, histology, and electron microscopy. They found significant fatty infiltration, increased interstitial connective tissue, and a sevenfold decrease in elasticity relative to controls.^109^ These physiologic changes initially worsened after repair but, importantly, never improved to prerepair values.^109^ Building off this work, this group next performed a pharmacologic intervention study in a rabbit model of rotator cuff tear: when ND was administered (systemically and/or locally) at the time of supraspinatus release, these animals demonstrated significantly less supraspinatus retraction and no fatty infiltration compared with controls with supraspinatus release alone.^40^ This was the first work to show partial prevention of musculotendinous changes after rotator cuff release using AASs.

In a separate experiment, the same group found that AAS administration did not contribute to muscle regeneration in a sheep model of a chronic rotator cuff tear.^41^ Taken together with the previous study, this suggests that AASs may be effective in preventing degenerative muscle changes in the rotator cuff but are not regenerative.^40,41^ Most recently, the authors showed that AASs given at the time of a rotator cuff repair can prevent additional muscle atrophy but not fatty infiltration, which suggests that the beneficial effects of AASs may depend on how quickly they are administered after rotator cuff injury.^39^ The translational potential of these findings is currently under investigation: an ongoing randomized controlled trial (RCT) at the University of Southern California aims to examine the potential role of oral oxandrolone to facilitate healing of repaired rotator cuff tendons and to improve the functional outcomes in patients with chronic, degenerative rotator cuff tears who undergo arthroscopic repair (NCT03091075).^118^

### B. Improved Postoperative Recovery

#### B1. Hip Fracture Repair

Hip fractures are a common cause of morbidity and mortality in older people.^119^ It is estimated that up to 50% of women and nearly 25% of men are at risk for an osteoporotic fracture in their lifetime.^120^ These patients tend to be frail and undernourished and may have poor mobility at baseline related to reduced muscle mass and strength. Despite surgery and rehabilitation, many patients experience a further decline in mobility and function, which has significant personal and societal costs.^121^ Early mobility has been found to be predictive of improved outcomes.^119^ As AASs have had positive effects in treating other catabolic states, it is plausible that AASs may improve perioperative recovery and outcomes in the hip fracture population.

Although the overall quality of the evidence is low, a few small RCTs have provided mixed results on the effect of AAS on functional outcome after hip fracture repair in older patients (Table 1).^37,122-124^ In a pilot trial of 31 frail elderly females (mean age 82 years), Sloan et al.^122^ found no difference in the number of patients upgraded to a higher level of care, deaths, time to independent mobilization, or incidence of adverse events between patients who received postoperative nandrolone injections (weekly for up to 4 weeks) and those receiving placebo. A different study randomized 23 patients (aged >60 years) recovering from hip fracture surgery, and administered varied doses of AASs dependent on sex, and measured blood testosterone concentration.^124^ The authors noted no significant differences in knee extension strength between the AAS group and placebo at the final (14 weeks) follow-up. In another trial of 40 lean elderly women, Tidermark et al.^37^ compared AAS injections every 3 weeks for 6 months and daily protein supplementation compared with protein supplementation alone. The authors found some evidence, albeit weak, that AAS use was associated with positive effect on lean body mass and patient-reported function and quality of life.^37^ In a post hoc analysis of the same trial, Tengstrand et al.^125^ found that AASs did not seem to have any additional effect on BMD measured by dual-energy X-ray absorptiometry (DEXA) scan compared with protein-rich supplementation alone.

**Table 1:** Clinical Studies Examining the Potential Benefit of Perioperative Use of Androgenic Anabolic Steroids to Accelerate Patient Recovery

Study	Level of Evidence	Type of Surgical Procedure	Study Design	Participants	Intervention	AAS Administration Route	AAS Dose	Duration of AAS Administration	Latest Postoperative Follow-up Time
Sloan et al.^119^	I	Hip fracture	RCT	Women aged >65 yr undergoing surgical fixation of hip fracture (N = 31)	Postoperative ND versus placebo	IM	2 mg/kg administered weekly	4 wk or until hospital discharge, whichever came first	4 wk or until hospital discharge, whichever came first
Tidermark et al.^34^	I	Hip fracture repair	RCT	Women aged >70 yr with BMI <24 kg/m^2^ undergoing surgical fixation of femoral neck fracture, independent walking/living status (N = 60)	Postoperative ND + protein-rich formula + daily calcium and vitamin D versus protein-rich formula alone versus daily calcium and vitamin D alone	IM	25 mg administered every 3 wk	6 mo	12 mo
Hedstrom et al.^120^	I	Hip fracture repair	RCT	Women aged >65 yr undergoing surgical fixation of hip fracture, independent living status (N = 63)	Postoperative ND + daily calcium and vitamin D versus daily calcium only	IM	25 mg administered every 3 wk	12 mo	12 mo
Hulsbæk et al.^124^	I	Hip fracture repair	RCT	Adults aged >60 yr admitted to the hip fracture unit (n = 23)	Postoperative ND + protein-rich nutritional drinks vs placebo + protein-rich nutritional drinks	IM	Female: 50 mg; males: 100 mg or 200 mg dependent on total testosterone level every 3 wk	14 wk	14 wk
Wu et al.^32^	I	ACL reconstruction	RCT	Otherwise healthy men aged 18-50 yr undergoing ACL reconstruction (N = 13)	Perioperative testosterone versus placebo, weekly IM testosterone 200 mg injections versus placebo. Injections began 2 wk before surgery to 6 wk postoperatively	IM	200 mg administered weekly	8 wk, beginning 2 wk before surgery and continuing until 6 wk after surgery	12 wk
Michelsen et al.^124^	II	THA	RCT	Otherwise healthy adults undergoing THA (N = 17)	Postoperative ND versus placebo	IM	200 mg administered as a single postoperative injection	Single dose	Postoperative day 4
Amory et al.^125^	I	TKA	RCT	Otherwise healthy men aged >55 yr undergoing TKA (N = 25)	Preoperative testosterone enanthate versus placebo injections	IM	600 mg administered at 21, 14, 7, and 1 d before surgery	4 wk	5 wk
Hohmann et al.^33^	I	TKA	RCT	Otherwise healthy adults aged 50-70 yr undergoing TKA (N = 10)	Postoperative ND versus placebo	IM	50 mg administered every 2 wk starting on postoperative day 5	6 mo	12 mo

AAS = anabolic androgenic steroid, ACL = anterior cruciate ligament, BMI = body mass index, ND = nandrolone decanoate, THA = total hip arthroplasty, TKA = total knee arthroplastyI, M = Intramuscular, RCT = Randomized Controlled Trial

In contrast, a separate trial in elderly women (mean age 80.5 years) found that AAS injections every 3 weeks for 1 year and daily supplementation with vitamin D and calcium were associated with higher BMD, gait speed, and Harris Hip Scores after hip fracture compared with supplementation with calcium alone.^123^ While the dose and frequency of ND injections in this trial was the same as those in Tidermark, the differences in duration (1 year versus 6 months, respectively), and nutritional supplementation (calcium and vitamin D versus protein, respectively) makes it difficult to directly compare the results of these studies.^37,123^

Overall, the small size and heterogeneous study designs of current trials provide insufficient data to draw conclusions on the effects of AASs on functional outcomes after hip surgery.^121^ As a recent Cochrane review notes, “Given that the available data points to the potential for more promising outcomes with a combined anabolic steroid and nutritional supplement intervention, we suggest that future research should focus on evaluating this combination.”^121^

#### B3. Anterior Cruciate Ligament Reconstruction

Testosterone may serve a role in ligament homeostasis and strength, as discussed previously.^53,72,73^ However, exogenous testosterone use has never been studied in the context of improving ligament healing after injury, such as in the case of ACL reconstruction (Table 1). Still, the anabolic effects of AAS on muscle may improve postoperative recovery from knee injuries such as ACL tears. Knee injuries are associated with rapid disuse atrophy of the affected leg, which is worsened by surgical trauma and knee immobilization in the postoperative period.^30,126^ These factors contribute to a lengthy rehabilitative process but even with rehabilitation many patients do not return to preinjury activity levels.^30,126^

Preoperative rehabilitation mitigates loss of muscle mass and has been associated with a quicker return to sport.^127^ A recent clinical trial by Wu et al.^35^ evaluated whether testosterone supplementation would similarly minimize muscle loss in the leg after ACL reconstruction. Although the sample size (n = 13 males) precludes definitive conclusions, the authors found that testosterone-treated male patients had increased lean body mass relative to controls at 6 weeks post-ACL reconstruction.^35^ There were no differences in extensor muscle strength or clinical outcome scores between treatment groups, but the authors note that the study was underpowered to detect these differences.^35^ Additional studies are needed to determine the effect of perioperative testosterone on leg strength and outcomes after ACL reconstruction.

#### Total Joint Arthroplasty

Similar to the rationale described in the clinical scenarios above, short-term AAS use has also been studied in perioperative period of patients undergoing total joint arthroplasty (Table 1). An early study by Michelsen et al.^128^ reported that patients receiving large-dose nandrolone injection (200 mg) in the immediate postoperative period after total hip arthroplasty improved nitrogen balance and attenuated trauma-related myocyte amino acid changes. In a pilot clinical trial, Amory et al.^129^ investigated the effect of preoperative testosterone on inpatient functional recovery in patients undergoing total knee arthroplasty. Patients pretreated with four weeks of testosterone (600 mg intramuscularly, weekly) exhibited a trend toward shorter hospital stay and improvements in walking and stair climbing during inpatient rehabilitation.^129^

A pilot clinical trial published by Hohmann et al.^36^ in 2010 described 10 patients given nandrolone or a sham injection biweekly for 6 months after TKA. Despite the small sample size, the group treated with nandrolone exhibited greater quadriceps muscle strength at 3, 6, and 12 months postoperatively.^36^ In addition, patients from the AAS group performed better across all postoperative functional testing, although only differences in the Knee Society Score at 6 weeks, 6 months, and 12 months reached statistical significance.^36^ Notably, the nandrolone group demonstrated decreased femoral and lumbar bone density, although these results were not statistically significant.^36^ As many patients undergoing TKA exhibit quadriceps deconditioning due to years of pain and inactivity, and the goal of TKA is to improve functional mobility, AAS may prove to be a useful adjunct in the perioperative period.

## Summary

AASs have shown great promise as potential therapeutic tool in a variety of clinical scenarios relevant to orthopaedic surgeons. Based on current evidence, AASs can augment biological healing after muscle injury, fracture repair, or rotator cuff repair and have the potential to improve postoperative recovery after ACL reconstruction or total joint arthroplasty. To realize the clinical potential of AASs in orthopaedic surgery, substantial efforts are needed in the preclinical and clinical arenas to better characterize their effects on tissues and establish optimized regimens.
